# Three New Cytotoxic Steroidal Alkaloids from *Sarcococca hookeriana*

**DOI:** 10.3390/molecules23051181

**Published:** 2018-05-15

**Authors:** Kang He, Jinxi Wang, Juan Zou, Jichun Wu, Shaojie Huo, Jiang Du

**Affiliations:** 1 The Key Laboratory of Miao Medicine of Guizhou Province, Guiyang College of Traditional Chinese Medicine, Guiyang 550025, Guizhou, China; hekang0851@163.com (K.H.); zoujuan@hotmail.com (J.Z.); wujichun2018@sina.com (J.W.); 15038115764@163.com (S.H.); 2 The Ethnical Medicine Research Institute of Qian Dong Nan Miao and Dong Autonomous Prefecture, Kaili 556000, Guizhou, China; 15185660328@163.com

**Keywords:** *Sarcococca hookeriana*, sarchookloides A–C, steroidal alkaloid, cytotoxicity

## Abstract

Three new steroidal alkaloids with an unusual 3*α* tigloylamide group, named sarchookloides A–C (**1**–**3**), were isolated along with four known compounds (**4**–**7**) from the roots of *Sarcococca hookeriana*. Their structures and relative configuration were elucidated on the basis of spectroscopic methods including MS, UV, IR, 1D, and 2D NMR data. The isolated compounds were evaluated for their cytotoxicity against five human cancer cell lines: Hela, A549, MCF-7, SW480, and CEM in vitro. All three amide substituted steroidal alkaloids exhibited significant cytotoxic activities with IC_50_ values of 1.05–31.83 μM.

## 1. Introduction

**Figure 1:**
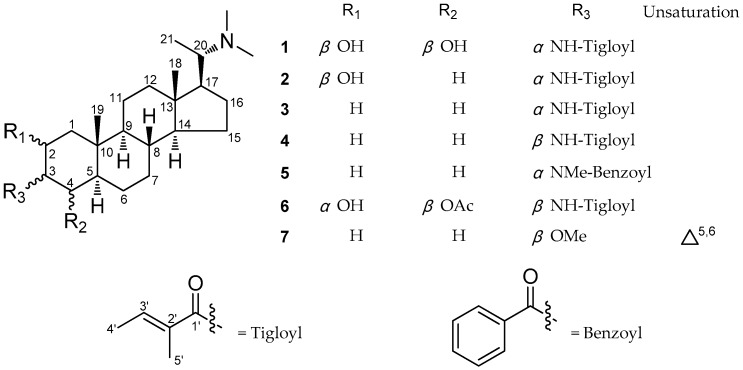
Structures of compounds **1**−**7**.

The genus *Sarcococca* (Buxaceae) includes about 20 species, eight of which are found in China [1]. Some of them are used in TCM and traditional folk medicine to treat stomach pain, rheumatism, swollen sore throat, and bruises [2,3,4]. Previous studies on this genus revealed that the steroidal alkaloids were the main chemical components, and possessed a range of bioactivities (e.g., cholinesterase inhibiting, antitumor, antibacterial, antiulcer, antiplasmodial, and antidiabetic) [5,6,7,8,9,10,11,12,13,14,15,16,17,18,19,20]. For the search of bioactive metabolites from this genus, our previous investigation on S*arcococca ruscifolia* resulted in the discovery of two new steroidal alkaloids [16]. As part of our continuous exploration of active alkaloids, three new steroidal alkaloids, namely sarchookloides A–C (**1**–**3**) along with four known compounds, pachysamine G (**4**), pachysamine H (**5**), sarcovagine B (**6**), and pachyaximine A (**7**) (Figure 1), were isolated from the roots of *Sarcococca hookeriana*. The new compounds, sarchookloides A–C (**1**–**3**), were shown to possess a 3*α* substituent, which has rarely been reported [17]. The cytotoxicity assay on human cancer cell lines Hela, A549, MCF-7, SW480, and CEM in vitro demonstrated that these steroidal alkaloids exhibited potent antitumor activities. This paper describes the isolation, structure elucidation, and cytotoxicity activities of the isolates.

## 2. Results and Discussion

### 2.1. Structure Elucidation of Compounds

Compound **1** showed a quasi-molecular ion peak [M + H]^+^ at *m*/*z* 461.3731 (calculated to be 461.3738) in the HR-ESI-MS (spectrum showed in Supplementary material), which corresponds to the molecular formula C_28_H_48_N_2_O_3_. The IR spectrum showed absorption bands at 3424 (hydroxyl group), 1662 (amide carbonyl group), and 1623 (double bond) cm^–1^. The ^1^H and ^13^C NMR (DEPT) spectra (Table 1) displayed 28 carbon resonances due to four quaternary carbons, 10 methines, seven methylenes, and seven methyl groups, which revealed one amide carbonyl group and one double bond. The presence of five methyl signals [*δ*_H_ 0.67 (3H, s, H-18), 1.21 (3H, s, Me-19), 0.92 (3H, d, *J* = 6.4 Hz, Me-21), 2.23 (6H, s, *N*,*N*-dimethyl)] and one nitric proton signal [*δ*_H_ 6.09 (1H, d, *J* = 5.1 Hz, NH-3)] in the ^1^H NMR (CDCl_3_) spectrum in combination with 2D NMR data suggested that compound **1** belongs to the 20*α*-dimethylamino-3-amino-5*α*-pregnane type steroidal alkaloids [21].

**Figure 2:**
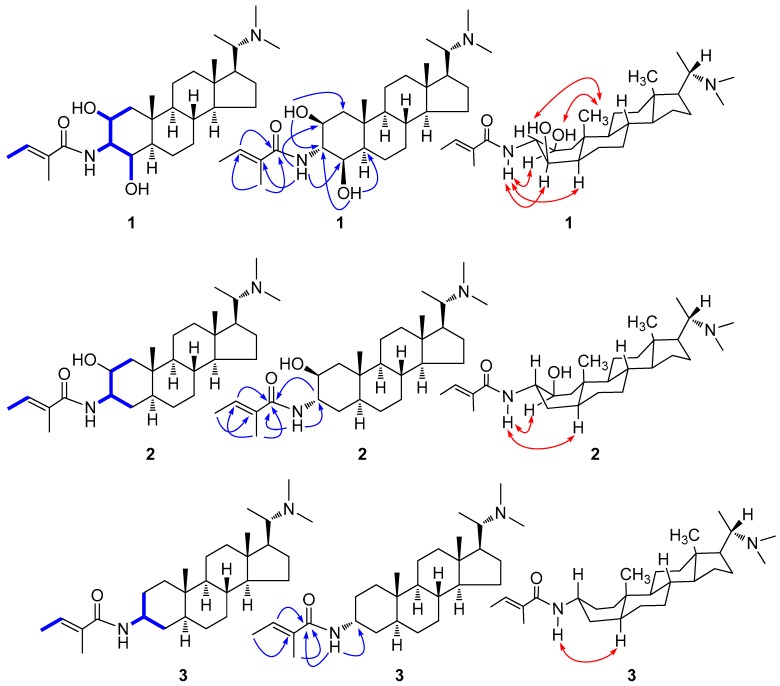
Key 1H−1H COSY (‒), HMBC (**→**) and ROESY (**↔**) correlations of compounds **1**–**3**.

The presence of two methyl [*δ*_H_ 1.76 (3H, d, *J* = 6.9 Hz, H-4′), 1.84 (3H, s, H-5′)] and an olefinic proton [*δ*_H_ 6.45 (1H, q, *J* = 6.9 Hz, H-3′)] signals in the ^1^H NMR spectrum together with two methyl [*δ*_C_ 14.3 (C-4′), 12.6 (C-5′)], one double bond [*δ*_C_ 131.4 (C-2′), 132.1 (C-3′)], and one carbonyl [*δ*_C_ 170.6 (C-1′)] signals in the ^13^C NMR spectrum led to the deduction of a tigloyl moiety, which was supported by the ^1^H–^1^H COSY-correlated signal of H-4′/H-3′ and the HMBC correlations of H-5′/C-3′, C-1′ and H-3′/C-1′ (Figure 2). Furthermore, the HMBC correlations of NH/C-2′ (Figure 2) proposed that the location of the tigloyl group was at N-3. In addition, the two hydroxyl groups assigned at the C-2 (*δ*_C_ 69.0) and C-4 (*δ*_C_ 76.3) positions were deduced from the ^1^H NMR [*δ*_H_ 2.84 (1H, d, *J* = 7.2 Hz, OH-2), 4.40 (1H, d, *J* = 3.0 Hz, OH-4)] and ^13^C NMR [*δ*_C_ 69.0 (C-2), 76.3 (C-4)] data in combination with the ^1^H–^1^H COSY [OH-2 /H-2 (*δ*_H_ 3.88) and OH-4 /H-4 (*δ*_H_ 3.78)] and HMBC [OH-2/C-1 (*δ*_C_ 44.2), C-3 (*δ*_C_ 58.4) and OH-4/C-3, C-5 (*δ*_C_ 45.5)] experiments. Therefore, the planar structure of compound **1** was constructed.

The ^13^C NMR data of the ring A**,** coupling constants of H-2, H-3 and H-4 and NOESY data clearly indicated that compound **1** differs from the previously reported compounds of hookerianamide M [10] and sarcovagine A [18] with respect to the stereochemistry at C-2, C-3, and C-4 positions. In the ROESY spectrum (Figure 2), the correlations of H-19 [*δ*_H_ 1.21 (3H, s)] with OH-2 and OH-4 indicated the *β* orientations of the two hydroxyl groups. Furthermore, the obvious ROESY correlations (Figure 2) of HN with H-2, H-4 and H-5 [*δ*_H_ 1.26 (1H, m)] implied the *α* orientation of the tigloylamide group. The ring A of compound **1** may exist mainly as a stable boat conformation due to the substitution of 3*α* tigloylamide group [17]. Consequently, the structure and relative configuration of compound **1** was determined as (20*S*)-20-*N*,*N*-dimethylamino-2*β*,4*β*-dihydroxyl-3*α*-tigloylamino-5*α*-pregnane, which was named sarchookloide A (Figure 1).

Compound **2** had a molecular formula of C_28_H_48_N_2_O_2,_ which was determined by HR-ESI-MS (*m*/*z* 445.3775 [M + H]^+^), suggesting six degrees of unsaturation. The IR spectrum displayed absorptions indicating a hydroxyl group (3443 cm^–1^), amide carbonyl group (1664 cm^−1^) and double bond (1629 cm^−1^). In the ^13^C NMR and DEPT spectra (Table 1), 28 carbon signals were observed, including 23 carbon resonances assigned to a 20*α*-dimethylamino-3-amino-5*α*-pregnane type steroidal alkaloid skeleton and 5 carbon resonances for a tigloyl group [21]. The ^1^H–^1^H COSY correlated signal of H-4′ [*δ*_H_ 1.75 (3H, d, *J* = 6.9 Hz)]/H-3′ [*δ*_H_ 6.37 (1H, q, *J* = 6.9 Hz)] and the HMBC correlations of H-5′ [*δ*_H_ 1.84 (1H, s)]/C-3′ [*δ*_C_ 130.8], C-1′ [*δ*_C_ 169.2], H-3′/C-1′ and HN [*δ*_H_ 5.82 (1H, d, *J* = 7.4 Hz)]/C-1′ (Figure 2) suggested that the tigloyl group were attached to N-3. The COSY correlations of H-2 [*δ*_H_ 3.97 (1H, brs)]/H-1 [*δ*_H_ 1.16, 1.87 (2H, m)], H-3 [*δ*_H_ 4.03 (1H, m)] proposed that the location of the hydroxyl group was at C-2.

The similarity of the NMR data of compounds **2** and 20*α*-dimethylamino-2*α*-hydroxyl-3*β*-tigloylamino-5*α*-pregnane [16] suggested that they possessed the same planar structure. The ROESY correlations (Figure 2) of HN with H-2 and H-5 [*δ*_H_ 1.07 (1H, m)] implied the *α* orientation of the tigloylamide group and the *β* orientation of the hydroxyl group. This was also supported by the discrepant W_1/2_ (14.8) of H-2 [10] and the ^13^C NMR data of the ring A in **2** compared with the data of reported compounds [16]. The substitution of 3*α* tigloylamide group led to the main boat conformation of the ring A in compound **2**. Consequently, the structure and relative configuration of compound **2** was determined as (20*S*)-20-*N*,*N*-dimethylamino-2*β*-hydroxyl-3*α*-tigloylamino-5*α*-pregnane, which was named sarchookloide B (Figure 1).

Compound **3** was given the molecular formula C_28_H_48_N_2_O according to its HR-ESI-MS data at *m*/*z* 429.3829 [M + H]^+^ (calculated as 429.3839), corresponding to six degrees of unsaturation. The IR spectrum of compound **3** included the absorption bands for amide carbonyl group (1665 cm^−1^) and double bond (1625 cm^−1^). The ^13^C NMR and DEPT spectra (Table 1) of compound **3** exhibited 28 carbon signals corresponding to four quaternary carbons, eight methines, nine methylene, and seven methyl groups, of which 23 carbon resonances were ascribed to 20*α*-dimethylamino-3-amino-5*α*-pregnane type steroidal alkaloid skeleton and five carbon resonances were attributed to a tigloyl group [21].

Compound **3** and pachysamine G [19] exhibited the same planar structure, which was supported by the similar NMR data found for both of them. The *α*-orientations of the tigloylamide group was assigned by the ROESY correlations (Figure 2) of HN [*δ*_H_ 5.93 (1H, d, *J* = 6.6 Hz)] with H-5 [*δ*_H_ 1.11 (1H, m)] in combination with the different shift of the ring A in compound **3** compared with the data of pachysamine G in ^13^C NMR spectrum. Similar to compounds **1** and **2**, the boat conformation of the ring A in compound **3** was the main conformation [17]. Therefore, the structure and relative configuration of compound **3** was determined as (20*S*)-20-*N*,*N*-dimethylamino-3*α*-tigloylamino-5*α*-pregnane, which was named sarchookloide C (Figure 1).

The known compounds **4**–**7** were identified as pachysamine G (**4**) [19], pachysamine H (**5**) [19], sarcovagine B (**6**) [18], and pachyaximine A (**7**) [20] through a comparison of their spectroscopic data with those reported in the literature.

### 2.2. Results of the Cytotoxicity Test

All compounds were evaluated using a MTT cytotoxicity assay against human cervical cancer cell line Hela, lung adenocarcinoma cell line A549, breast cancer cell line MCF-7, colon cancer cell line SW480, and leukemia CEM cells (adriamycin was used as the positive control). The IC_50_ values of all compounds against the indicated cancer cells are summarized in Table 2. Compound **5** had the greatest cytotoxicity to all cells, as its range of IC_50_ values was approximately 1.05–2.23 μM. All three amide-substituted compounds, except for pachyaximine A, exhibited significant cytotoxic activity on all cells, which suggests that the amide group of these compounds was the necessary group for the cytotoxicity. In addition, Hela and A549 were the more sensitive cell lines to these types of compounds compared to all tested cancer cells because the IC_50_ values of all compounds were less than 10 μM. Furthermore, all active compounds showed effects that were comparable to the chemotherapeutic drug adriamycin in inhibiting the growth of all cancer cells, which suggests that three amide-substituted pregnane-type steroidal alkaloids might have the potential to be anticancer agents.

## 3. Materials and Methods

### 3.1. General Experimental Procedures

Optical rotations were obtained on a JASCO model 1020 polarimeter (Horiba, Tokyo, Japan). UV spectra were measured on a Shimadzu UV-2401PC spectrophotometer (Shimadzu, Kyoto, Japan). IR (KBr) spectra were measured on a Bio-Rad FTS-135 spectrometer (Bio-Rad, Hercules, CA, USA). The 1D and 2D NMR spectra were recorded on Bruker AVANCE III-600 spectrometers with TMS used as an internal standard (Bruker, Bremerhaven, Germany). The mass spectra were obtained on a Waters AutoSpec Premier P776 (Waters, NY, USA). The silica gel (200–300 mesh) for column chromatography and the TLC plates (GF_254_) were obtained from Qingdao Marine Chemical Factory (Qingdao, Shandong, China). The Sephadex LH-20 (20–150 μm) used for chromatography was purchased from Pharmacia Fine Chemical Co. Ltd. (Pharmacia, Uppsala, Sweden). Fractions were visualized by heating silica gel plates sprayed with Dragendorff’s reagent. The cell lines Hela, A549, MCF-7, SW480, and CEM were obtained from the Cell Bank of the Chinese Academy of Sciences (Shanghai, China), while MTT were obtained from Sigma Company.

### 3.2. Plant Material

The plants of *Sarcococca hookeriana* Baill. were collected in Hezhang County, Guizhou Province, China, in April 2012 and identified by Prof. Qingwen Sun, Guiyang College of Traditional Chinese Medicine. A voucher specimen (No. 20120401401) was deposited at the Key Laboratory of Miao Medicine of Guizhou Province, Guiyang College of Traditional Chinese Medicine.

### 3.3. Extraction and Isolation

The air-dried and powdered roots of *S*. *hookeriana* Baill. (2.5 kg) were extracted with 95% (25 L) EtOH under reflux three times, with an extraction time of 2 h. The combined extracts (443 g) were concentrated and suspended in H_2_O (3 L). The suspension was extracted with CHCl_3_ to obtain the CHCl_3_ fraction (94 g). The CHCl_3_ fraction was subjected to silica gel column chromatography (Si CC) and eluted with petroleum ether-diethylamine (100:1, 95:5, 9:1, 8:2) to yield four fractions (Fractions A−D). Fraction B (1.6 g) was subjected to Si CC and eluted with petroleum ether-diethylamine (100:2, 9:1) to yield the fractions B1−B3. Fraction B2 (330 mg) was chromatographed using Si CC and was developed with petroleum ether-diethylamine (100:2) to yield compounds **3** (21 mg) and **4** (35 mg). Si CC was performed on Fraction C (12.3 g) with a gradient eluent of petroleum ether-diethylamine (100:5, 9:1, 8:2) to yield four fractions (fractions C1−C4). Fraction C2 (1.4 g) was first subjected to Si CC (petroleum ether-diethylamine, 100:5), before being purified on Sephadex LH-20. This yielded compounds **2** (26 mg), **5** (30 mg) and **7** (31 mg). Fraction C4 (985 mg) was purified on Sephadex LH-20, before Si CC was used (petroleum ether-diethylamine, 9:1) to separate compounds **1** (33 mg) and **6** (19 mg).

#### 3.3.1. Sarchookloide A (**1**)

This was a white amorphous powder with a HR-ESI-MS *m*/*z* of 461.3731 [M + H]^+^ (calculated for C_28_H_49_N_2_O_3_, 461.3738). The [α]${}_{D}^{22}$ was +52.99 (*c* 0.58, MeOH); UV (MeOH) had a λ_max_ of 209.4 nm; and IR (KBr) had a ν_max_ of 3424, 2931, 2867, 1662 and 1623 cm^–1^. The ^1^H and ^13^C NMR data are shown in Table 1.

#### 3.3.2. Sarchookloide B (**2**)

This was a white amorphous powder with a HR-ESI-MS *m*/*z* of 445.3775 [M +H]^+^ (calculated for C_28_H_49_N_2_O_2_, 445.3789). The [α]${}_{D}^{22}$ was +30.18 (*c* 0.57, MeOH); UV (MeOH) had a λ_max_ of 205.8 nm; and IR (KBr) had a ν_max_ of 3442, 2930, 2966, 1664 and 1629 cm^–1^. The ^1^H and ^13^C NMR data are shown in Table 1.

#### 3.3.3. Sarchookloide C (**3**)

This was a white amorphous powder with a HR-ESI-MS *m*/*z* of 429.3829 [M + H]^+^ (calculated for C_28_H_49_N_2_O, 429.3839). The [α]${}_{D}^{22}$ was +7.10 (*c* 0.62, MeOH); UV (MeOH) had a λ_max_ of 207.2 nm; and IR (KBr) had a ν_max_ of 3454, 2930, 2853, 1665 and 1625 cm^–1^. The ^1^H and ^13^C NMR data are shown in Table 1.

### 3.4. Cytotoxicity Assay

The cytotoxicity of compounds **1**–**7** was tested on the human cervical cancer cell line Hela, lung adenocarcinoma cell line A549, breast cancer cell line MCF-7, colon cancer cell line SW480 and leukemia CEM cells. All cells were cultured in a RPMI-1640 or DMEM medium (Hyclone, Logan, UT, USA), which was supplemented with 10% fetal bovine serum (Hyclone) in 5% CO_2_ at 37 °C. The cytotoxicity assay was performed using the MTT method in 96-well microplates [22]. Briefly, the adherent cells (100 μL) were seeded into each well of 96-well cell culture plates and allowed to adhere for 12 h before the addition of the drug. The suspended cells were seeded just before the addition of the drug at an initial density of 1 × 10^5^ cells/mL. Each tumor cell line was exposed to the tested compound at different concentrations for 48 h. The experiments were performed in triplicate. Adriamycin (Sigma, St. Louis, MO, USA) was used as a positive control. After treatment, cell viability was measured and the cell growth curve was plotted. The IC_50_ values were calculated by the Reed and Muench method [23].

## 4. Conclusions

We obtained three new pregnane-type steroidal alkaloids, sarchookloides A–C (**1**–**3**), along with four known compounds, pachysamine G (**4**), pachysamine H (**5**), sarcovagine B (**6**), and pachyaximine A (**7**), from the roots of *Sarcococca hookeriana*. The new compounds, sarchookloides A–C (**1**–**3**), were shown to possess a 3*α* substituent, which has rarely been reported. By performing a cytotoxic assay on Hela, A549, MCF-7, SW480 and CEM cell lines in vitro, all three amide substituted compounds exhibited significant cytotoxic activities on all cells, which suggests that the three amide group of these compounds was the necessary group for the cytotoxicity. The most active compound, pachysamine H (**5**), inhibited all cancer cells with IC_50_ values in the range of approximately 1.05–2.23 μM. The results suggested that these types of steroidal alkaloids merit further biological evaluation of their cytotoxic activities and might have the potential to be studied for anticancer activity.

## Figures and Tables

**Table 1:** ^1^H (600 MHz) and ^13^C (150 MHz) NMR data of compounds **1**−**3** in CDCl_3_.

Position	1		2		3	
	*δ*_H_ (*J* in Hz)	*δ*_C_	*δ*_H_ (*J* in Hz)	*δ*_C_	*δ*_H_ (*J* in Hz)	*δ*_C_
1	1.67, 1.69, m	44.2	1.16, 1.87, m	40.6	0.95, 1.56, m	33.6
2	3.88, ddd (13.4, 6.7, 6.8)	69.0	3.97, brs (W_1/2_ 14.8)	69.6	1.64, 1.71, m	26.2
3	3.99, ddd (7.8, 6.8, 5.1)	58.4	4.03, m	50.8	4.14, m	44.8
4	3.78, dd, (7.8, 3.9)	76.3	1.23, 2.04, m	28.8	1.39, 1.55, m	33.0
5	1.26, m	45.5	1.07, m	41.8	1.11, m	41.5
6	1.41, 1.79, m	24.4	1.48, 1.85, m	27.9	1.48, 1.87, m	27.8
7	1.01, 1.77, m	32.2	0.91, 1.68, m	32.1	0.88, 1.68, m	32.1
8	1.37, m	35.3	1.39, m	34.9	1.36, m	35.5
9	0.71, m	57.0	1.05, m	56.8	0.68, m	54.7
10	-	36.0	-	35.9	-	36.2
11	1.35, 1.44, m	21.0	1.30, 1.50, m	21.0	1.22, 1.51, m	20.9
12	1.12, 1.90, m	39.9	1.09, 1.90, m	40.0	1.08, 1.88, m	40.0
13	-	42.1	-	41.8	-	41.5
14	1.03, m	56.8	0.66, m	55.6	1.04, m	56.8
15	1.10, 1.60, m	24.2	1.06, 1.58, m	24.2	1.03, 1.57, m	24.2
16	1.50, 1.84, m	27.9	1.35, 1.44, m	28.4	1.19, m	28.6
17	1.34, m	54.8	1.32, m	54.8	1.33, m	54.7
18	0.67, s	12.6	0.64, s	12.6	0.63, s	12.5
19	1.21, s	19.3	1.02, s	14.5	0.79, s	11.6
20	2.50, m	61.6	2.45, m	61.6	2.45, m	61.6
21	0.92, d, (6.4)	10.2	0.89, d, (6.4)	10.2	0.88, d, (6.4)	10.3
NMe2	2.23, s	39.8	2.20, s	39.9	2.20, s	39.9
C=O	-	170.6	-	169.2	-	168.8
2′	-	131.4	-	132.2	-	132.6
3′	6.45, q, (6.9)	132.1	6.37, q, (6.9)	130.8	6.36, q, (6.9)	130.2
4′	1.76, d, (6.9)	14.3	1.75, d, (6.9)	14.2	1.73, d, (6.9)	14.1
5′	1.84, s	12.6	1.84, s	12.7	1.83, s	12.7
NH	6.09, d, (5.1)	-	5.82, d, (7.4)	-	5.93, d, (6.6)	-
2-OH	2.84, d, (7.2)	-	-	-	-	-
4-OH	4.40, d, (3.0)	-	-	-	-	-

**Table 2:** Cytotoxicity of compounds **1**−**7** ^a^ against Hela, A549, MCF-7, SW480, and CEM cells in vitro (IC_50_^b^, μM).

Compounds	Cell Lines				
	Hela	A549	MCF-7	SW480	CEM
**1**	4.13 ± 0.14	2.53 ± 0.15	4.47 ± 0.06	6.42 ± 0.10	4.26 ± 0.11
**2**	7.93 ± 0.09	8.73 ± 0.16	28.53 ± 0.17	8.97 ± 0.10	31.83 ± 0.25
**3**	1.24 ± 0.10	2.87 ± 0.14	2.53 ± 0.12	3.08 ± 0.14	3.43 ± 0.13
**4**	2.43 ± 0.11	2.98 ± 0.17	3.70 ± 0.26	26.04 ± 0.21	3.05 ± 0.13
**5**	1.06 ± 0.14	1.18 ± 0.11	2.23 ± 0.15	1.49 ± 0.10	1.05 ± 0.06
**6**	1.38 ± 0.09	4.96 ± 0.12	1.65 ± 0.09	3.76 ± 0.14	6.06 ± 0.16
**7**	>100	>100	>100	>100	>100
Adriamycin ^c^	0.62 ± 0.08	0.77 ± 0.06	1.26 ± 0.05	1.19 ± 0.11	0.98 ± 0.08

^a^ All results are expressed as mean ± SD; *n* = 3 for all groups. ^b^ IC_50_: 50% inhibitory concentration. ^c^ Adriamycin was the positive control.

## Supplementary Materials

The following ^1^H NMR, ^13^C NMR, 2D NMR, HR-ESI-MS spectra and the RAW data of the new compounds are available as supporting data. Supplementary materials are available online.
