# Supplementary material for: Three New Cytotoxic Steroidal Alkaloids from Sarcococca hookeriana
Source: Molecules. 2018 May 15;23(5):1181. doi: 10.3390/molecules23051181 (PMC6099570; doi:10.3390/molecules23051181)
Supplement: Supplementary file 1 [file molecules-23-01181-s001.zip › Supporting Information/Supporting Information.doc]

**Supporting Information**

Three new cytotoxic steroidal alkaloids from *Sarcococca hookeriana*

Kang He ^1,†^, Jinxi Wang ^2,†^, Juan Zou ^1^, Jichun Wu ^1^, Shaojie Huo ^1^ and Jiang Du ^1,^*

^1^	The Key Laboratory of Miao Medicine of Guizhou Province, Guiyang College of Traditional Chinese Medicine, Guiyang 550025, Guizhou, China; hekang0851@163.com (K.H.); zoujuan@hotmail.com (J.Z.); 493808408@qq.com (J.W.); 871977797@qq.com (S.H.)

^2^	The Ethnical Medicine Research Institute of Qian Dong Nan Miao and Dong Autonomous Prefecture, Kaili 556000, Guizhou, China; 923206756@qq.com (J.W.)

*****	Correspondence: dujang.gz@163.com (J.D.); Tel.: +86-871-88308060 (J.D.)

^†^	These authors contributed equally to this work and are co-first authors.

**Supporting Information List**

Characterization Data of New Compounds **1**−**3**

**►** ^1^H NMR, ^13^C NMR, DEPT, HSQC, HMBC, COSY, NOESY and HR-ESI-MS spectra of sarchookloid A (**1**)

**►** ^1^H NMR, ^13^C NMR, DEPT, HSQC, HMBC, COSY, NOESY and HR-ESI-MS spectra of sarchookloid B (**2**)

**►** ^1^H NMR, ^13^C NMR, DEPT, HSQC, HMBC, COSY, NOESY and HR-ESI-MS spectra of sarchookloid C (**3**)

**For sarchookloid A (1):**

**Spectra 1.** ^1^H NMR spectrum of sarchookloid A (**1**)

**Spectra 2.** ^13^C NMR and DEPT spectrum of sarchookloid A (**1**)

**Spectra 3.** HSQC spectrum of sarchookloid A (**1**)

**Spectra 4.** HMBC spectrum of sarchookloid A (**1**)

**Spectra 5.** COSY spectrum of sarchookloid A (**1**)

**Spectra 6.** ROESY spectrum of sarchookloid A (**1**)

**Spectra 7.** HR-ESI-MS spectrum of sarchookloid A (**1**)

**For** sarchookloid B **(2)**

**Spectra 8.** ^1^H NMR spectrum of sarchookloid B **(2)**

**Spectra 9.** ^13^C NMR and DEPT spectra of sarchookloid B **(2)**

**Spectra 10.** HSQC spectrum of sarchookloid B **(2)**

**Spectra 11.** HMBC spectrum of sarchookloid B **(2)**

**Spectra 12.** COSY spectrum of sarchookloid B **(2)**

**Spectra 13.** ROESY spectrum of sarchookloid B **(2)**

**Spectra 14.** HR-ESI-MS data of sarchookloid B **(2)**

**For** sarchookloid C **(3)**

**Spectra 15.** ^1^H NMR spectrum of sarchookloid C **(3)**

**Spectra 16.** ^13^C NMR and DEPT spectra of sarchookloid C **(3)**

**Spectra 17.** HSQC spectrum of sarchookloid C **(3)**

**Spectra 18.** HMBC spectrum of sarchookloid C **(3)**

**Spectra 17.** HSQC spectrum of sarchookloid C **(3)**

**Spectra 18.** HMBC spectrum of sarchookloid C **(3)**

**Spectra 19.** COSY spectrum of sarchookloid C **(3)**

**Spectra 20.** ROESY spectrum of sarchookloid C **(3)**

**Spectra 21.** HR-ESI-MS data of sarchookloid C **(3)**
